# Biological subtypes of Alzheimer disease: A systematic review and meta-analysis

**DOI:** 10.1212/WNL.0000000000009058

**Published:** 2020-03-10

**Authors:** Daniel Ferreira, Agneta Nordberg, Eric Westman

**Affiliations:** From the Division of Clinical Geriatrics (D.F., A.N., E.W.), Department of Neurobiology, Care Sciences and Society, Karolinska Institutet, Huddinge, Sweden; Theme Aging (A.N.), Karolinska University Hospital, Huddinge, Sweden; and Department of Neuroimaging (E.W.), Centre for Neuroimaging Sciences, Institute of Psychiatry, Psychology and Neuroscience, King's College London, London, United Kingdom.

## Abstract

**Objective:**

To test the hypothesis that distinct subtypes of Alzheimer disease (AD) exist and underlie the heterogeneity within AD, we conducted a systematic review and meta-analysis on AD subtype studies based on postmortem and neuroimaging data.

**Methods:**

EMBASE, PubMed, and Web of Science databases were consulted until July 2019.

**Results:**

Neuropathology and neuroimaging studies have consistently identified 3 subtypes of AD based on the distribution of tau-related pathology and regional brain atrophy: typical, limbic-predominant, and hippocampal-sparing AD. A fourth subtype, minimal atrophy AD, has been identified in several neuroimaging studies. Typical AD displays tau-related pathology and atrophy both in hippocampus and association cortex and has a pooled frequency of 55%. Limbic-predominant, hippocampal-sparing, and minimal atrophy AD had a pooled frequency of 21%, 17%, and 15%, respectively. Between-subtype differences were found in age at onset, age at assessment, sex distribution, years of education, global cognitive status, disease duration, APOE ε4 genotype, and CSF biomarker levels.

**Conclusion:**

We identified 2 core dimensions of heterogeneity: typicality and severity. We propose that these 2 dimensions determine individuals' belonging to one of the AD subtypes based on the combination of protective factors, risk factors, and concomitant non-AD brain pathologies. This model is envisioned to aid with framing hypotheses, study design, interpretation of results, and understanding mechanisms in future subtype studies. Our model can be used along the A/T/N classification scheme for AD biomarkers. Unraveling the heterogeneity within AD is critical for implementing precision medicine approaches and for ultimately developing successful disease-modifying drugs for AD.

Alzheimer disease (AD) is a heterogeneous disease. Variability in age at onset and clinical presentation is known since the very first reports. Extensive research showed marked differences between early-onset and late-onset AD, mostly with early-onset AD showing more pronounced pathologic findings such as more tau-related pathology and brain atrophy.^1^ Regarding variation in the clinical presentation, different clinical subtypes have extensively been investigated, showing that the amnestic presentation is more common than nonamnestic presentations such as posterior cortical atrophy (PCA), logopenic primary progressive aphasia (LPPA), and the frontal variant of AD.^2^ Of interest, an association between age at onset and clinical presentation exists, with nonamnestic subtypes having earlier onset.^1^

Recent progress in biomarker research and wider availability of postmortem data has intensified the study of biologically defined subtypes of AD (based on neuropathologic and neuroimaging data). Several studies have been published including subtypes based on postmortem data, MRI data, and recently, tau-PET data. With the new definition of AD as a biological disease,^3^ it is now timely and unprecedented to review the literature on biologically defined subtypes of AD. We conducted a systematic review on the topic and calculated meta-analytical estimates aiming at characterizing the AD subtypes across key demographic and clinical measures. Our ultimate goal was to advance in our understanding of mechanisms driving heterogeneity in AD, hopefully contributing to guide the search for subtype-specific therapies within the current efforts of precision medicine.

## Methods

### Search strategy and selection criteria

This study followed the PRISMA statement. We conducted a systematic review using EMBASE, PubMed, and Web of Science in July 2019 without any limits in publication dates. The search strategy combined the following medical subject heading and free-text terms (data available from Dryad, table e-1, doi.org.10.5061/dryad.h70rxwdf3): “Alzheimer,” “AD,” “subtype,” “heterogeneity,” “atrophy,” “patterns,” “subtypes,” “MRI,” “Magnetic Resonance,” “PET,” “postmortem,” “neurofibrillary tangle,” and “neuropathological”. Additional relevant publications were identified by scrutinizing references of the included articles.

Selection criteria for the meta-analysis included (1) case-control studies reporting summary estimates on subgroups of patients with AD based on grouping strategies applied on MRI, PET, or postmortem data; (2) availability of data on subtypes frequency and key demographic and clinical measures; and (3) studies published in English.

Study selection was performed by a single researcher (D.F.), involving a second researcher (E.W.) when needed. When the selected studies included data from the same cohort, the studies were included in the meta-analysis if the grouping strategy differed across studies. When the grouping strategy was the same, the study with larger sample or reporting more subtypes was included. When data were missing for a variable, the next larger study was included (data available from Dryad, table e-2, doi.org.10.5061/dryad.h70rxwdf3).

Several strategies were followed to reduce risks bias related to publication, data availability, and reviewer selection (data available from Dryad, table e-3, doi.org.10.5061/dryad.h70rxwdf3).

### Data analysis

Data were collected for the fields listed in data available from Dryad (table e-4, doi.org.10.5061/dryad.h70rxwdf3). Data extraction was performed by a single researcher (D.F.). Studies' methodological quality was assessed with the CASP checklist for case control studies.^4^ Variables included in the meta-analysis were subtypes frequency (group size), age, sex, years of education, MMSE, CDR sum of boxes (CDR-SOB), age at onset, disease duration, APOE genotype, and CSF levels of amyloid-beta 1–42 (Aβ_42_), total tau (T-tau), and phosphorylated tau (p-tau). Using random-effects models, weighted pooled means (*p*M) and proportions (*p*P) were calculated for each subtype, and post hoc paired comparisons were performed with a *p* value <0.05 deemed significant. Heterogeneity in these analyses was investigated through visual inspection of forest plots and by computing the I^2^ parameter. *p* Values, 95% CI, and other meta-analytical parameters are provided in table 2 and data available from Dryad (figures e-1–e-28): doi.org.10.5061/dryad.h70rxwdf3. Analyses were conducted in R version 3.2.4 (R Core Team, 2014), using the “Meta” package.^5^

### Data availability

Data not provided in the article because of space limitations can be shared at request.

## Results

**Figure 1:**
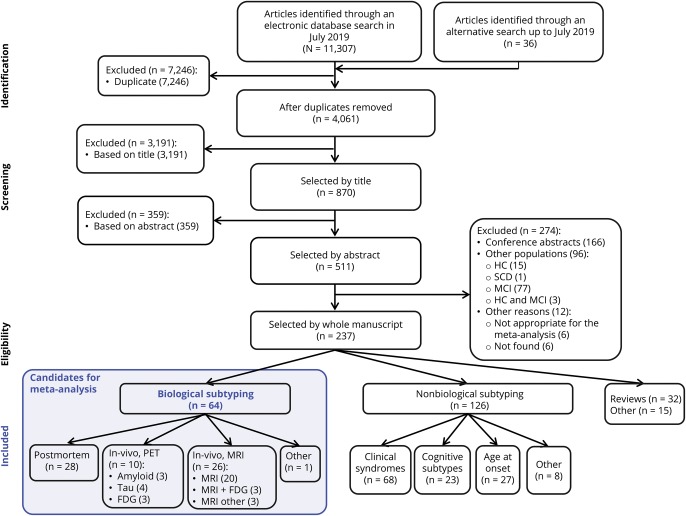
Study selection flowchart A total of 64 records were considered as candidates for the meta-analysis. Of those, 40 records were excluded because of the reasons listed in data available from dryad table e-5, doi.org.10.5061/dryad.h70rxwdf3, and the remaining 24 studies were included in the meta-analysis. FDG = fluorodeoxyglucose; HC = healthy control; MCI = mild cognitive impairment; SCD = subjective cognitive decline.

A total of 11,307 records were identified in the initial search. After removing duplicates and screening by title, abstracts, and full text, 64 records were selected (figure 1). Of those, we further excluded 40 records because of the reasons listed in data available from Dryad, table e-5, doi.org.10.5061/dryad.h70rxwdf3. This gave a total of 24 studies for the meta-analysis. Table 1 shows some characteristics of these studies (see data available from Dryad, table e-6, doi.org.10.5061/dryad.h70rxwdf3, for an extended description). All the selected studies had an appropriate methodological quality according to the CASP checklist.

**Table 1:**
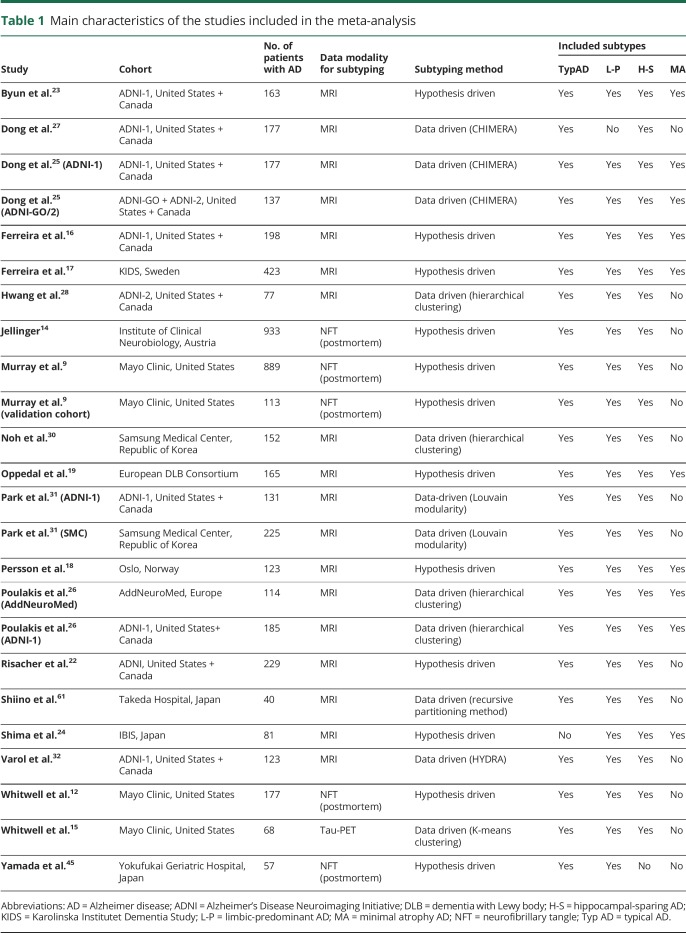
Main characteristics of the studies included in the meta-analysis

Our review shows that both hypothesis-driven and data-driven methods have been used for determining biological subtypes of AD. A number of early studies^6–8^ used principal component analysis and other clustering methods on pathologic measures of senile plaques, neurofibrillary tangles (NFTs), and cerebral amyloid angiopathy (CAA). Later studies mainly focused on markers of tau pathology and neurodegeneration. A seminal study investigated 889 brain autopsies and identified 3 AD subtypes based on the distribution of NFT: typical AD, with balanced NFT counts in the hippocampus and association cortex; limbic-predominant AD, with counts predominantly in hippocampus; and hippocampal-sparing AD, with counts predominantly in the association cortex.^9^ In that study, subtyping was based on a hypothesis-driven strategy in which regions of interest were a priori defined (hippocampus and 3 cortical association regions). The subtypes were identified according to patients' distribution on the hippocampus-to-cortex NFT spectrum by separating the groups at 25th and 75th percentiles. The same method was used in later postmortem studies from the same^10,11^ and other independent postmortem series.^9,12–14^ These subtypes have also been found in neuroimaging studies. Recently, the first study using tau-PET for subtyping was published^15^ and reported 3 subtypes likely corresponding to the postmortem subtypes. In addition, the postmortem subtypes can be reliably tracked in vivo by investigating patterns of atrophy in structural MRI.^12^ Hence, neurodegeneration measures are another plausible vein to defining biological subtypes. Several hypothesis-driven MRI studies have reported data on these subtypes by using visual rating scales of brain atrophy^16–21^ or automated methods for estimating regional brain volume.^22,23^ MRI studies have also consistently identified a fourth subtype displaying minimal brain atrophy.^16–21,23–26^ In contrast, data-driven methods do not depend on a priori selection of brain regions but investigate interindividual variation in measures of volume or cortical thickness. Several algorithms have been used.^15,25–33^ Recently, the first subtyping study using diffusion tensor imaging for white matter integrity was published.^34^ Our systematic review did not identify any data-driven study on FDG-PET, another imaging modality for measuring neurodegeneration. We identified 3 hypothesis-driven studies comparing patients with AD with different patterns of FDG-PET uptake (see data available from Dryad table e-5, doi.org.10.5061/dryad.h70rxwdf3).

**Figure 2:**
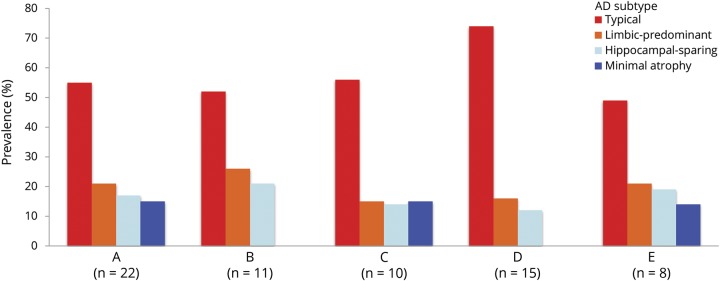
Frequency of the AD subtypes The frequency of the subtype partially depends on the number of subtypes included in each study. The figure shows frequency estimates for all the studies pooled together (A, n = 22); studies including 3 subtypes (generally typical, limbic-predominant, and hippocampal-sparing AD) (B, n = 11); and studies including 4 subtypes (C, n = 10). Other factors that may influence these estimates are the modality of the data subtyping is performed on such as postmortem vs MRI data, as well as the subtyping method. The seminal subtyping algorithm^9^ is used in all the postmortem studies and in 1 MRI study,^22^ and frequency values relate to the 25th and 75th percentiles applied on the hippocampus-to-cortex NFT/atrophy ratio (D, n = 15). Studies not using this algorithm use a variety of subtyping methods and include mostly MRI studies, except for a tau-PET study^15^ and a postmortem study,^45^ both excluded from this subanalysis) (E, n = 8). AD = Alzheimer disease; NFT = neurofibrillary tangle.

We calculated pooled estimates of the frequency of the subtypes. Typical AD is the most frequent subtype, with a pooled frequency of 55%. In contrast, limbic-predominant, hippocampal-sparing, and minimal atrophy AD had a pooled frequency of 21%, 17%, and 15%, respectively (table 2). The frequency of the subtypes partially depended on factors such as the number of subtypes included in each study, the modality of the data subtyping is performed on (e.g., postmortem vs MRI), and the algorithm used for subtyping (figure 2).

**Table 2:**
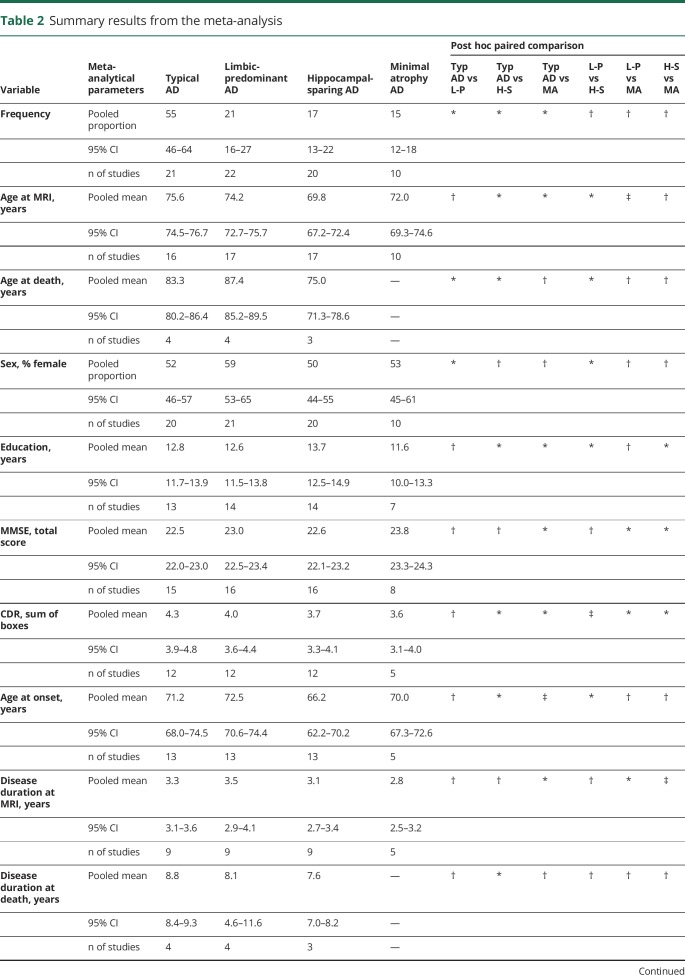

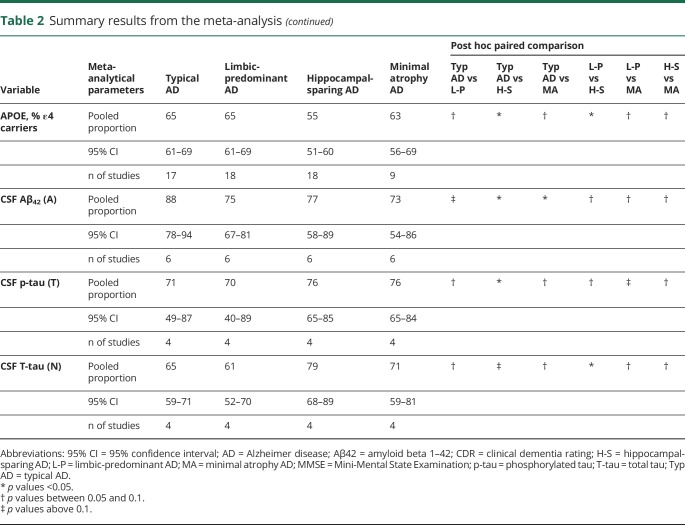
Summary results from the meta-analysis

We also calculated meta-analytical estimates aiming at characterizing the subtypes across key demographic and clinical measures. Results are shown in table 2 and figure 2. Forest plots and the I^2^ parameter are shown in data available from Dryad, figures e-1–e-28, doi.org.10.5061/dryad.h70rxwdf3. Typical and limbic-predominant were significantly older than hippocampal-sparing and minimal atrophy AD. Limbic-predominant AD included the highest frequency of females. Hippocampal-sparing included highly educated individuals, whereas minimal atrophy AD included less educated individuals. Minimal atrophy AD had better MMSE and CDR scores than the other subtypes. Hippocampal-sparing had earlier disease onset compared with typical and limbic-predominant AD. Minimal atrophy AD had the shortest disease duration. Hippocampal-sparing included a lower frequency of APOE ε4 carriers compared with typical and limbic-predominant AD. Hippocampal-sparing had less amyloid and more tau-related pathology than typical and had more neurodegeneration (i.e., CSF total tau) than limbic-predominant AD. The I^2^ parameter indicated high heterogeneity in several models (see forest plots and the I^2^ parameter in data available from Dryad, figures e-1–e-28, doi.org.10.5061/dryad.h70rxwdf3).

## Discussion

We conducted a systematic review and meta-analyses of studies including biologically defined subtypes of AD. We identified 3 dimensions that are major drivers of the heterogeneity within AD: (1) risk factors, including sex, APOE, and age; (2) protective factors, including aspects such as cognitive reserve, brain resilience, and brain resistance; and (3) concomitant non-AD pathologies that together with amyloid (A) and tau-related pathology (T) may contribute to the neurodegeneration (N) seen in AD, which are formulated under the A/T/N framework for AD.^35^

The current meta-analysis revealed that hippocampal-sparing AD had the earliest disease onset, and typical and limbic-predominant AD had the latest disease onset. No significant differences were found for minimal atrophy AD, which showed an intermediate age at onset. Because the 4 subtypes do not differ substantially in disease duration, the age at the time of MRI is the lowest in hippocampal-sparing AD and the highest in typical and limbic-predominant AD, with minimal atrophy AD in between. The ADNI cohort mostly included patients with late-onset amnestic AD, and therefore, the hippocampal-sparing subtype found in ADNI studies does not completely correspond to the group of patients with early-onset hippocampal-sparing AD seen in other cohorts. Regarding postmortem studies, our meta-analysis shows that the age results are slightly different because unlike MRI studies usually including younger cohorts with patients at mild to moderate dementia stages, postmortem studies often include older cohorts at advanced stages of the disease. Hence, the age at death in postmortem studies is the oldest for limbic-predominant AD and the youngest for hippocampal-sparing AD, reflecting that hippocampal-sparing is the most aggressive form, with faster progression, and younger age at death,^29^ whereas limbic-predominant AD has a later onset and slower disease progression. This finding is coherent with the possible contribution of TAR DNA-binding protein 43 (TDP-43) pathology, hippocampal-sclerosis, and the microtubule-associated protein tau (MAPT) H1H1 genotype to limbic-predominant AD, 3 factors that are related to atrophy restricted to the medial temporal lobes, older age, and slower disease progression.

Female patients more frequently had limbic-predominant AD, and male patients more frequently had hippocampal-sparing AD. We also found that APOE ε4 carriers more frequently have limbic-predominant and typical AD, whereas APOE ε4 noncarriers more frequently have hippocampal-sparing AD. APOE ε4 has consistently been associated with lower volume in hippocampus and adjacent regions.^36,37^ This finding was observed also in neonates, whereas APOE ε4 noncarrier neonates had lower volume in parietal, frontal, and occipital cortices.^38^ This finding in neonates may predispose APOE ε4 carriers and noncarriers to different regional brain vulnerability later in life. A finding supporting this APOE genotype-specific vulnerability is that APOE ε4 carriers have higher tau-PET binding in the entorhinal cortex, whereas APOE ε4 noncarriers have higher tau-PET binding in parietal and occipital cortices.^36^ Therefore, APOE ε4 negative may increase resistance of the hippocampus to accumulate tau pathology, but would in turn increase vulnerability of posterior brain areas to accumulate tau pathology. This supports the theory that the absence of the APOE ε4 allele may drive pathology toward posterior brain areas^36^ or, alternatively, expose the effect of pathologies affecting posterior brain areas commonly masked by APOE ε4.^2^ These masked pathologies may include cerebrovascular disease (CVD) and Lewy body pathology (discussed later in this article).

The strong connection between sex, APOE, and age has been coined as “the triad of risk of AD”.^37^ Below we propose that this triad together with other factors drives the distribution of pathology seen in limbic-predominant and hippocampal-sparing AD, diverging from that seen in typical AD.

Genetic research in AD subtypes has also focused on the MAPT H1H1 genotype. The frequency of the MAPT H1H1 genotype is increased in limbic-predominant AD compared with typical and hippocampal-sparing AD.^9,10^ However, a study reported that typical AD had the highest frequency of MAPT H1H1 carriers, followed by limbic-predominant and hippocampal-sparing AD.^22^ These findings suggest that the MAPT H1H1 genotype may play a role in the medial temporal lobe predilection for NFT.

Coexisting with risk factors such as female sex, APOE ε4, and older age, there are counteracting protective forces such as cognitive reserve and the related concepts of brain resilience/resistance.^39,40^ For example, higher cognitive reserve has been associated with lower cortical tau-PET binding,^41^ which has been interpreted as brain resistance in the sense that higher cognitive reserve may contribute to avoid tau aggregation.^40^ Another example is that higher cognitive reserve has been associated with lower hippocampal volume in amyloid positive cognitively normal individuals,^42^ which is commonly interpreted as brain resilience, i.e., higher cognitive reserve contributes to cope with neurodegeneration better.^40^ However, the presence of 1 APOE ε4 allele can reduce the protective effects of education,^43^ a proxy of cognitive reserve.

How risk and protective factors balance and cancel each other out is not completely understood. Multiple pathways may exist, which would eventually increase the heterogeneity within AD. Our meta-analysis revealed clear differences in education across subtypes. Hippocampal-sparing AD has the highest level of education, whereas minimal-atrophy AD has the lowest level of education. This finding is observed in the context of similar levels of clinical severity: the 4 subtypes had rather comparable scores on MMSE and CDR-SOB. We acknowledge that statistically significant differences were revealed for both MMSE and CDR-SOB. However, the differences did not exceed more than 1 point in any of these scales, which probably lack clinical relevance. A previous study supports this interpretation showing comparable levels of impairment in activities of daily living across subtypes.^22^ Why hippocampal sparing has higher levels of education is not known. We suggest that high education could be one of the factors protecting the hippocampus so that pathologies affecting the posterior cortex and thus contributing to hippocampal-sparing AD may be unmasked and have a greater contribution to the clinical expression of the disease.

The concepts of cognitive reserve, brain resilience/resistance connect directly with brain pathology, in this case, amyloid, tau-related pathology, and neurodegeneration. The theory of cognitive reserve postulates that people with higher reserve can cope with brain pathology better.^39^ At similar levels of clinical severity, the cognitive reserve theory would foresee more pathology in individuals with higher reserve and less pathology in individuals with less reserve. The finding of more intense neurodegeneration and more aggressive disease progression in hippocampal-sparing,^9,10,18,23,29^ the subtype with the highest level of reserve, supports this hypothesis. The absence of overt brain atrophy in minimal atrophy AD, the subtype with the lowest level of reserve, also supports this hypothesis. The big question is which pathology or pathologies are patients with minimal atrophy AD so vulnerable to? In a previous study, we observed that the frequency of abnormal CSF p-tau and T-tau levels was higher in minimal-atrophy AD than in the other subtypes.^17^ Based on that observation, we proposed that tau-related pathology and neurodegeneration at the molecular level may be sufficient to give dementia symptoms in patients with minimal atrophy AD in the absence of overt brain atrophy.^17,44^ Our meta-analysis provides preliminary support to that explanation by showing higher frequency of abnormal CSF p-tau and T-tau levels in minimal atrophy compared with typical and limbic-predominant AD. However, these comparisons did not yield statistical significance, perhaps due to low statistical power.

In a recent tau-PET study,^15^ limbic-predominant AD had focal tau-PET binding bilaterally in the temporal lobes (medial, inferior, and middle temporal areas), extending to the posterior cingulate. Both patients with typical and hippocampal-sparing AD showed tau-PET binding throughout the temporal, parietal, occipital, and frontal lobes, with greater binding in typical AD.

Apart from CSF T-tau, neurodegeneration has also been investigated through FDG-PET, which at the moment is much more clinically established. Hippocampal-sparing AD displayed greater hypometabolism in prototypic AD regions than typical and limbic-predominant AD.^22^ Two other studies showed that hypometabolism matches the patterns of brain atrophy that defines the subtypes.^24,28^ Of interest, patients with minimal-atrophy AD also showed reduced metabolism in the parietal cortex despite lacking overt brain atrophy in those regions.^24^ Below we elaborate on how this finding may be related to network disruption in minimal-atrophy AD.^21^

All the postmortem studies included in this meta-analysis^9–14,45^ as well as the tau-PET study^15^ and 1 MRI study^22^ exclusively recruited amyloid-positive patients with AD. Our meta-analysis on CSF Aβ_42_ levels showed that typical AD displayed the highest proportion of amyloid-positive patients (88%), whereas the other 3 subtypes displayed comparable proportions including around 75% of the patients. The proportion of amyloid-positive patients in typical AD differed significantly from hippocampal-sparing and minimal-atrophy AD, whereas it reached a trend for statistical significance when compared with limbic-predominant AD. Primary studies showed no differences in the global amount of senile plaques or global amyloid PET binding across subtypes.^10,12,22^ However, a study reported greater global amyloid PET binding in patients with limbic-predominant AD.^15^ In contrast, regional analyses have revealed several differences. Typical AD has higher counts of senile plaques in occipital regions compared with limbic-predominant AD.^9^ Hippocampal-sparing AD showed marked amyloid PET binding in frontal and parietal cortices compared with that in typical and limbic-predominant AD.^28^ Furthermore, limbic-predominant AD showed greater amyloid PET binding in medial frontal and parietal cortices compared with that in typical AD. Nonetheless, the distribution of amyloid pathology in the brain is rather diffuse, which perhaps explains why we could only find 1 study drawing AD subtypes exclusively from amyloid measures.^46^

AD pathology rarely occurs in isolation. Patients with AD pathology at autopsy often have concomitant pathologies such as CVD, Lewy body pathology, and/or TDP-43.^47,48^ How non-AD brain pathologies contribute to the heterogeneity within AD, perhaps underlying the biological subtypes reviewed here, is a topic of great interest. The scarce amount of data at the time being prevented us to conduct specific meta-analytical calculations.

The contribution of vascular factors has been investigated in several studies. Hypertension was reported to be more frequent in typical and limbic-predominant AD,^31^ whereas no differences were reported in another study.^18^ Furthermore, the subtypes seem to be rather comparable in the frequency of other vascular risk factors such as diabetes, dyslipidemia, and cardiovascular disease.^18,31^ Regarding CVD, postmortem series from the Mayo Clinic showed increased CVD in typical and limbic-predominant AD.^9,10,12,13^ However, postmortem series from the Institute of Clinical Neurobiology in Vienna reported increased CVD only in typical AD.^14^ The burden of white matter hyperintensities (WMHs) in MRI is higher in typical and limbic-predominant AD.^25^ We recently conducted a comprehensive characterization of the AD subtypes in terms of CVD, including the amount and distribution of deep/lobar microbleeds and WMH, cortical superficial siderosis, perivascular spaces, lacunes, large brain infarction, and intracerebral hemorrhage.^17^ CAA seemed to make a stronger contribution to hippocampal-sparing and minimal atrophy, whereas hypertensive arteriopathy may make a stronger contribution to typical and limbic-predominant AD.

Other non-AD brain pathologies have received less attention. In the report by Josephs et al.,^13^ hippocampal sclerosis was more frequent in limbic-predominant than in typical and hippocampal-sparing AD, although this observation did not reach statistical significance. Furthermore, hippocampal sclerosis was associated with TDP-43 deposition only in patients with limbic-predominant AD.^13^ The strong association between hippocampal sclerosis and TDP-43 has been reported several times.^11^ TDP-43 is increased in typical and limbic-predominant compared with hippocampal-sparing AD.^9,10,13^ This finding converges with previous studies, showing that the amygdala is the first and most commonly affected region by TDP-43 deposition^49^ and that TDP-43 is strongly associated with smaller hippocampal volume.^50^ Of interest, clinical presentation in AD seems to be driven by subtype, not by TDP-43.^13^ Hippocampal sclerosis, TDP-43, and limbic-predominant AD are more common in older women with a slowly progressive amnestic syndrome.^10^ The clinical entity LATE (limbic-predominant age-related TDP-43 encephalopathy) has recently emerged.^51^

The MAPT H1H1 genotype may play a role in the medial temporal lobe predilection for NFT. To our knowledge, no previous studies have investigated the association among MAPT H1H1 genotype, hippocampal sclerosis, and TDP-43.

Despite the clear clinical differentiation between dementia with Lewy bodies (DLBs) and AD, the comorbidity between the 2 is high.^47,48^ The postmortem series from the Mayo Clinic in Jacksonville showed increased Lewy body pathology in typical and limbic-predominant AD.^9,10^ In contrast, the postmortem series from Vienna and Mayo Clinic in Rochester reported increased Lewy body pathology in hippocampal-sparing AD.^12–14^ Cholinergic dysfunction is central in DLB and AD. We recently investigated whether the cholinergic basal forebrain is differentially affected and influences different treatment response across AD subtypes.^20^ We found that the volume of the cholinergic basal forebrain declines more rapidly in typical and limbic-predominant AD, whereas treatment response seemed to be better in hippocampal-sparing AD.^20^ Dong et al.^27^ also reported reduced volume of the basal forebrain in 2 AD subtypes likely resembling typical and limbic-predominant AD. Because patients with DLB (whose most common pattern of brain atrophy is hippocampal sparing^19^) and patients with AD with less hippocampal atrophy respond well to cholinesterase inhibitors,^52,53^ we suggest that the interplay between pattern of atrophy and the cholinergic system may be an explanation for good response to cholinergic treatment in DLB and hippocampal-sparing AD. Whether a common pattern of brain atrophy or increased Lewy body pathology in hippocampal-sparing AD or both is the reason for this finding needs to be elucidated. The dopaminergic system is heavily involved in DLB, whereas it is spared in AD, which could also explain marginal differences in the response to cholinesterase inhibitors.

All these concomitant brain pathologies may be especially harmful to individuals with low cognitive reserve such as patients with minimal-atrophy AD. Tau-related pathology and neurodegeneration at the molecular level may be sufficient to disrupt key brain networks, which could give the symptoms in the absence of overt brain atrophy in minimal-atrophy AD.^21^ The finding of reduced metabolism in the parietal cortex in minimal atrophy AD^24^ supports the explanation regarding network disruption. Increased small vessel disease in posterior white matter in minimal atrophy AD^17^ could also have an impact on such networks. An almost identical pattern of brain hypometabolism and small vessel disease colocalizes with reduced white matter integrity in diffusion tensor imaging and correlates with cognitive impairment in the absence of overt brain atrophy in DLB.^54–57^ Of interest, minimal atrophy was the second most common pattern in DLB.^19^ Therefore, tau and concomitant pathologies may disconnect key brain networks in minimal atrophy AD, giving the symptoms in the absence of overt brain atrophy. We showed that 3 networks involving posterior brain regions (fronto-parietal, visual, and default-mode networks) are affected in minimal-atrophy AD.^21^

Atypical nonamnestic presentations such as PCA, LPPA, and the frontal variant of AD are more common in hippocampal-sparing than in typical and limbic-predominant AD.^9,12,13,15^ Some cohorts showed virtually no differences in the cognitive profile across subtypes.^16,18,24,28^ Other cohorts showed greater impairment of nonmemory functions in hippocampal-sparing AD.^30,31^ Cognitive trajectories over time more clearly differ across subtypes, highlighting heterogeneity in disease progression as well. Longitudinal decline in MMSE was faster in hippocampal-sparing than in the other subtypes.^9,10,23,29^ Na et al.^29^ showed that this finding extends to several memory and nonmemory cognitive domains that also decline faster in hippocampal-sparing AD. However, other studies showed that typical AD has the fastest longitudinal decline in MMSE.^16,18^ The results on longitudinal decline in CDR are similar than those for MMSE, that is, faster decline is reported for typical AD in a study,^16^ whereas faster decline is reported for hippocampal-sparing AD in other studies.^18,29^ These contradictory results may depend on differences across cohorts in the frequency of patients with early-onset nonamnestic AD presentations. Regarding psychiatric features, the subtypes have comparable levels of depressive^18,23,28^ and neuropsychiatric symptoms.^18,23^

This study has some limitations. Different methods have been used in the literature to define the subtypes. This methodological variability together with characteristics of the cohorts may be the explanations for the high heterogeneity in several of our models, as reflected by the I^2^ parameter in figures e-1–e-28, doi.org.10.5061/dryad.h70rxwdf3. We conducted random-effect models to partially account for this problem, but the heterogeneity seen in this meta-analysis may have an impact on some of the pooled estimates. Hence, we call for the urgent need to harmonize subtyping methods across studies. Another limitation is that some analyses included few studies due to lack of data at present, and some factors could not be analyzed. Therefore, we encourage that future studies should focus on CSF biomarkers or PET imaging and concomitant pathologies. Finally, most of the MRI studies in this meta-analysis included mixed cohorts of amyloid positive and negative patients. This may include heterogeneity not due to AD. Hence, it is important that future studies focus on amyloid-positive patients with AD and better characterize their participants, for instance, by using the A/T/N framework.

**Figure 3:**
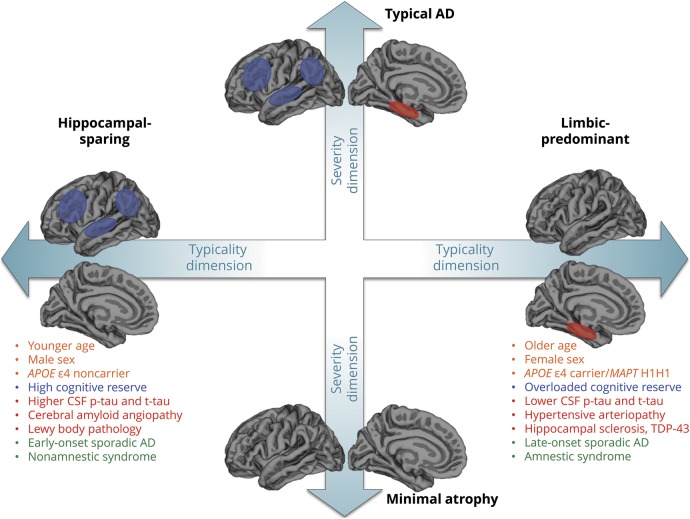
Framework for future studies on AD subtypes The figure represents 2 dimensions: typicality and severity. We propose that the combination of risk factors, protective factors, and diverse brain pathologies will determine individuals' location along the typicality and severity dimensions, giving 4 distinct subtypes: typical AD, limbic-predominant AD, hippocampal-sparing AD, and minimal atrophy AD. The blue and red ellipsoids on the brain representations show the regions defining these 4 subtypes according to previous studies.^9,23^ The figure also lists the risk factors, protective factors, and brain pathologies. In orange, the risk factors, including age, sex, and APOE. In blue, the protective factors, including cognitive reserve and related concepts such as brain resilience and brain resistance. In red, brain pathologies including AD pathologies and concomitant non-AD pathologies. AD pathologies can be organized using the A/T/N classification scheme.^35^ In this meta-analyses, characterization of A/T/N categories across subtypes was performed through CSF biomarkers (table 2): an amyloid biomarker should be positive in our model and its load is similar across subtypes, the reason why amyloid is not depicted in the figure; tau-related pathology was assessed with CSF phosphorylated tau (p-tau), and neurodegeneration was assessed with CSF total tau (t-tau). Concomitant non-AD pathologies include cerebrovascular disease (forms of small vessel disease such as cerebral amyloid angiopathy and hypertensive arteriopathy) and other pathologies such as Lewy body pathology, hippocampal sclerosis, and TDP-43. All these factors increase heterogeneity within AD and lead to subtypes according to the spread and location of pathology (neuropathologically and neuroimaging-defined subtypes) often aligning with clinically defined subtypes according to the age at onset and the cognitive presentation (in green in the figure). AD = Alzheimer disease.

In this systematic review, we identified 3 factors that may explain a substantial part of the heterogeneity within AD, that is, risk factors, protective factors, and concomitant non-AD brain pathologies. We propose that the balance between risk and protective factors determines brain regional vulnerability along the lifespan, contributing to differential spatial deposition of different pathologies and eventually leading to divergent clinicopathologic presentations of AD. Pursuing our goal of advancing our understanding of mechanisms driving heterogeneity in AD, we propose the model depicted in figure 3. We distinguish 2 main dimensions: typicality (with limbic-predominant on the one side and hippocampal-sparing on the other side, both deviating from typical AD in the middle) and severity (which emerges exclusively in neurodegeneration studies and separates minimal atrophy from typical AD). The severity dimension corresponds to the “N” category in the A/T/N classification scheme for AD biomarkers.^35^ The current meta-analysis contributes to better characterize the “N” category, adding a spatial dimension that includes different atrophy patterns connected with different factors that ultimately lead to distinct clinical manifestations of AD. The A and T categories are assumed to be positive in our model, adhering to the current biological definition of AD.^3^ Cognitive reserve and brain resilience/resistance levels would determine individuals' location along the severity dimension, with lower levels related to minimal atrophy AD. Future studies need to ascertain the distribution of NFT in minimal-atrophy AD. It is even possible that patients with minimal-atrophy AD have not yet reached the Braak stage V.^58^

Our proposal for explaining what determines individuals' location along the typicality dimension is as follows. The medial temporal lobes are a frequent site for deposition of various pathologies (i.e., tau aggregates, TDP-43, and hippocampal sclerosis) and are also more vulnerable to cerebrovascular pathology.^59^ It is possible that factors such as brain resilience help compensating for these pathologies until a certain level. However, a combination of risk factors might knock these compensatory effects out. This process takes time, which would explain the later-onset and slower progression of limbic-predominant AD, as well as why limbic-predominant AD is more common among females and APOE ε4 carriers. In the absence of these risk factors, brain resistance mechanisms would more efficiently preserve the medial temporal lobes and perhaps drive pathology to the posterior cortex or expose the effect of non-AD pathologies affecting the posterior cortex. This may explain the higher frequency of hippocampal-sparing AD in highly educated, young, APOE ε4 negative males, as well as higher frequency of CAA and Lewy body pathology in hippocampal-sparing AD. The explanation for early onset in hippocampal-sparing AD remains elusive, but the added onslaught of non-AD pathologies and increased vulnerability of key networks such as the default mode network are plausible explanations.

This meta-analysis and our proposed model highlight the importance of factors such as age, sex, and education as potential drivers of diverging pathophysiologic processes across subtypes. Therefore, these factors should be carefully considered, and whether they should be used as confounding variables is to be decided at the study level depending on the aims and characteristics of the cohorts.

We envision our proposed model as a framework to aid with framing hypotheses, study design, interpretation of results, and understanding mechanisms in future subtype studies. This framework can be used along the recently proposed A/T/N classification scheme for AD biomarkers.^35^ Here, we identify avenues for future research aiming at moving the field forward. Despite building on previous proposals to explain heterogeneity within AD,^2,60^ our current model offers a reformulation based on the findings accumulated during recent years. Our typicality and severity dimensions as well as recognition of key determinants such as compensatory processes are reminiscent of the 3 hypotheses proposed in a previous study,^60^ that is, the subtype model, phase model, and compensation model, respectively. Likewise, a previous model proposed 3 dimensions delineated by APOE, age at onset, and concomitant brain pathologies,^2^ which are integrated in our model as well. However, we believe that typicality (subtype model) and severity (phase model) dimensions cannot be teased apart as previously suggested^60^ and that modulatory factors (e.g., cognitive reserve, brain resilience/compensation model) are intricate with the other 2 dimensions. All these issues need to be recognized in an updated model; hence, they are integrated in our proposed framework.

We are still at the beginning of the long journey of completely unraveling the heterogeneity within AD. The extensive research on clinical heterogeneity accumulated during decades, together with the current availability of biological markers that open a window to mechanisms affecting the brain, will illuminate the way toward reaching the goals of precision medicine in the future. This is expected to get us closer to the ultimate goal of developing successful disease-modifying drugs for AD.

## Glossary

AD: Alzheimer disease
PCA: posterior cortical atrophy
LPPA: logopenic primary progressive aphasia
NFT: neurofibrillary tangle
CAA: cerebral amyloid angiopathy
MAPT: microtubule-associated protein tau
CVD: cerebrovascular disease
